# Mammotome^®^ biopsy system for the resection of breast lesions: Clinical experience in two high-volume teaching hospitals

**DOI:** 10.3892/etm.2013.1191

**Published:** 2013-07-01

**Authors:** YANGPING JIANG, HUANRONG LAN, QIAN YE, KETAO JIN, MIN ZHU, XIAOYAN HU, LISONG TENG, FEILIN CAO, XIANFANG LIN

**Affiliations:** 1 Department of Surgical Oncology, Dongyang Hospital, Wenzhou Medical University, Dongyang, Zhejiang 322100, P.R. China; 2 Department of Surgical Oncology, Wenzhou Medical University, Linhai, Zhejiang 317000, P.R. China; 3 Laboratory of Translational Oncology, Public Research Platform, Wenzhou Medical University, Linhai, Zhejiang 317000, P.R. China; 4 Department of Operation Room, Taizhou Hospital, Wenzhou Medical University, Linhai, Zhejiang 317000, P.R. China; 5 Department of Surgical Oncology, First Affiliated Hospital, Zhejiang University School of Medicine, Hangzhou, Zhejiang 310003, P.R. China; 6 Department of Ultrasonography, Taizhou Hospital, Wenzhou Medical University, Linhai, Zhejiang 317000, P.R. China

**Keywords:** vacuum-assisted biopsy, breast, benign breast lesions, ultrasound guidance, Mammotome biopsy

## Abstract

Ultrasound-guided vacuum-assisted breast biopsy (VABB) is regarded as a feasible, effective, minimally invasive and safe method for the removal of benign breast lesions, without the occurrence of serious complications. The aim of this study was to evaluate the feasibility, efficacy and safety of ultrasound-guided VABB using the Mammotome^®^ biopsy system in the treatment of breast lesions. The clinical outcomes of 3,681 patients with breast lesions were evaluated following excisions by ultrasound-guided VABB in two high-volume teaching hospitals. From January 2008 to December 2012, a total of 4,867 ultrasound-guided VABB procedures were performed in the 3,681 patients, who had a mean age of 37.8 years (range, 16–73 years). The parameters examined in this analysis included lesion size, lesion location in the inner breast, Breast Imaging Reporting and Data System (BI-RADS) ultrasound category and histopathological diagnosis. Ultrasonography follow-up was performed at 3–6 month intervals in order to assess recurrence. The size of the investigated lesions ranged between 6 and 62 mm and a histopathological diagnosis was made in 100% of cases. The results indicated that the majority of specimens (98.89%) were benign. On average, the ultrasound-guided VABB was performed in 10.3 min (range, 7.5–43 min) and the mean number of cores removed in the procedure was 8.1 (range, 3–32). A complete excision was achieved in the majority of cases (99.7%). The presence of a hematoma was the most common complication following the biopsy, and was observed in 27.5% of patients. The mean follow-up period was 25.5 months (range, 1–60 months), during which the rate of recurrence was 4.4%. The results indicated that ultrasound-guided VABB using the Mammotome biopsy system is an effective and safe procedure that is able to rapidly remove the majority of benign breast lesions using a small incision and without the occurrence of scarring or complications.

## Introduction

During the last two decades, the focus on the early detection of breast cancer has increased progressively, leading to the establishment of various breast cancer screening programs globally. Throughout the screening process, the number of suspicious findings on mammograms that are nonpalpable and nondetectable with ultrasound has increased markedly (1). The most common way to confirm the nature of the nonpalpable and sonographically occult findings on mammograms is minimally invasive breast biopsy.

With the development of minimally invasive breast biopsy systems, such as fine-needle aspiration cytology, core needle biopsy and vacuum-assisted biopsy, the diagnostic accuracy of breast lesions has been greatly improved (1). Large-bore, image-guided, vacuum-assisted biopsy has become recognized in recent years as a safe, cost-effective alternative to open surgery for the removal of certain benign breast lesions (2–4).

Although breast cancer is likely to affect one in eight females in their lifetime (5), current data estimate that 60% of all adult females are likely to acquire some form of benign breast disease. Moreover, up to 90% of clinical breast presentations are associated with a benign disease rather than malignancies (6). In general, females with benign breast disease are at no greater risk of developing breast cancer than other females (7–10), although certain studies have suggested a contrasting view (11,12). For those with benign breast disease, conservative treatment, including close surveillance, is the standard therapy. Although benign breast diseases are not life threatening, the existence of the abnormal lesions may arouse psychological stress and physical discomfort, such as pain, lumps or discharge in certain patients. In addition to causing anxiety for the patient, the lesions may grow over time, resulting in the ultimate removal of the lesions becoming increasingly challenging and less cosmetically pleasing (6). Therefore, in an increasing proportion of cases, the patients prefer for the lesions to be completely excised through a minimally invasive procedure. Following the excision, the final report concerning the pathology of the lesions may largely eliminate any concerns expressed by the patient. The preference for complete excision has also evolved due to the rapid progression in the development of minimally invasive diagnostic tools. Introduced in the late 1990s (13), the use of the Mammotome^®^ biopsy system (Ethicon Endo-Surgery, Inc., Cincinnati, OH, USA), an ultrasound-guided vacuum-assisted breast biopsy (VABB) system, has been suggested as a new strategy for breast lesions. With ultrasound-guided VABB, using the Mammotome biopsy system, benign breast lesions, including the surrounding normal tissue, are able to be excised in a minimally invasive manner; complete excision, without residual tissue is possible in the majority of cases (14). This has greatly prompted the complete excision of breast lesions in daily clinical practice.

The aim of this study was to assess the value of ultrasound-guided VABB in the treatment of breast lesions and to determine whether biopsy with a Mammotome biopsy system enabled resection to be avoided in patients with benign breast lesions.

## Patients and methods

### Patients

This study was approved by the Institutional Ethics Committees of the Dongyang (Dongyang, China) and Taizhou (Linhai, China) Hospitals of Wenzhou Medical University. In the period from January 2008 to December 2012, a total of 4,867 ultrasound-guided VABBs using the Mammotome biopsy system were performed in 3,681 patients, in the Departments of Surgical Oncology in Dongyang and Taizhou Hospitals, Wenzhou Medical University. All patients had a previously undergone a breast ultrasound, and for those over the age of 35 years, a mammography. The parameters analyzed included the size of the lesion, as shown in the mammogram or ultrasonogram, a peripheral or central location or a lump detected in a physical examination. Clinical data, including the Breast Imaging Reporting and Data System (BI-RADS) category, for the lesions were also obtained (15). None of the patients presented with discharge from the nipple. Taking into consideration the fact that the cost of ultrasound-guided VABB using the Mammotome system is not widely accepted in China, a therapeutic strategy was formulated. As such, ultrasound-guided VABB procedures were always performed for patients with a lesion(s) that was (were) most likely benign and that had a BI-RADS category of ≤4. By contrast, simple biopsy was permitted in cases of suspicious malignancy (BI-RADS category five) if the patient desired. In all cases, a preoperative core-needle gun biopsy was not performed prior to the ultrasound-guided VABB. Ultrasound-guided VABB was performed predominantly in patients who were expected to have a difficult follow-up and who had lesions of ≤3 cm that were classified as BI-RADS category three or four on ultrasonography, who were planning a pregnancy, who felt unease due to their lesions, whose lesions enlarged during follow-up and who complained of pains or other symptoms. In addition, ultrasound-guided VABB was performed in certain patients who refused to undergo surgical excision, despite presenting with lesions >3 cm, due to concern over breast scars. Informed consent was obtained from all the patients included in the present study. The patients who did not provide informed consent, who were allergic to the local anesthetic or who presented with active skin infections on the breast were disqualified from biopsy.

Bleeding during intervention, postinterventional hematoma and scar tissue formation were scored as small, moderate and severe. A small bleeding/hematoma was defined as a maximum aspiration of 20 ml blood or a maximum density area of 1.5×1.5×1.5 cm surrounding the target area in the postinterventional mammogram, respectively. A moderate bleeding/hematoma was defined as a maximum aspiration of 20–40 ml blood or a maximum density area of 3.0×3.0×3.0 cm, respectively, whereas a severe bleeding/hematoma was classified as an aspiration of >40 ml blood or a density area of >3.0×3.0×3.0 cm, respectively. The definition of small scar formation was a vague density, observed only along the z-axis of the biopsy probe, whereas moderate scar formation was defined as an area of density or an architectural distortion on one or both projection planes in the target area of the biopsy site. Severe scar formation was defined as a lesion causing diagnostic problems, regardless of the knowledge of previous biopsies, and thus necessitating additional mammography, ultrasound, re-biopsy or magnetic resonance imaging.

### Surgical procedure

Prior to the ultrasound-guided VABB procedure, the blood group and coagulation parameter examinations of the patients were evaluated. All the procedures were performed by two skilled surgeons and two ultrasound radiologists with experience of using the 8-gauge Mammotome biopsy system. A Terason t3000 ultrasound system (Teratech Corporation, Burlington, MA, USA) with high-resolution linear array transducers (12L5A, 5–12 MHz) was used to provide real-time ultrasound guidance.

Patients were maintained in a supine position with the ipsilateral arm raised above the head and with the target area sterilized and draped. An additional ultrasonic assessment was performed prior to the procedure. Following the administration of a local anesthetic consisting of 1% lidocaine with a 1:100,000 mixture of epinephrine, a 3–5 mm skin incision was made, which served as the access for the 8-gauge probe. Under real-time ultrasound guidance, the probe was positioned beneath the lesion. To ensure that the localization was accurate, the target lesion was rescanned longitudinally and transversely with the probe. During the procedure, the needle was rotated at an angle of 45° to either side, in order to completely excise the hypoechoic lesion on the intraoperative ultrasound and until normal fat tissue appeared on the core pieces. Any additional cores that were required were taken sequentially in different directions, also under ultrasound guidance, and a postprocedure sonographic evaluation was performed to confirm the complete excision. For hemostasis, direct compression was applied for 5–10 min immediately following the procedure. Subsequent to this, an elastic bandage was attached and the patient undertook 6 h bed rest.

A frozen section of the resected specimen was examined intraoperatively for pathological diagnosis. The tissue specimens were preserved in 10% formaldehyde solution and sent for histopathological evaluation to the Department of Pathology (Dongyang Hospital and Taizhou Hospital, Wenzhou Medical University). If malignancy was revealed, the appropriate management, such as chemotherapy or surgery, was performed; otherwise, the patient was able to return to daily life the day following the procedure. The follow-up was carried out with ultrasonography and mammography, at intervals of 3–6 months, in order to identify recurrences.

## Results

### Patient demographics and lesion parameters

A total of 4,867 consecutive 8-gauge ultrasound-guided VABB procedures were performed in 3,681 patients with a mean age of 37.8 years (range, 16–73 years). The data for parameters such as location in the inner breast, size of the lesion (as observed by ultrasonography and mammography), lesion characteristics and the BI-RADS category are presented in Table I. Six hundred and eighty two patients (18.5%) were ultrasonographically assessed to be BI-RADS category two, 2,150 (58.4%), category three, 801 (21.8%), category four and 48 (1.3%), category five (Table IA). On average, the procedure had a duration of 10.3 min (range, 7.5–43.0 min) and the mean number of cores removed in the procedure was 8.1 (range, 3–32). A complete excision was achieved in the majority of cases (99.7%). The average number of lesions in the patient population was 1.41 (range, 1–11). The size of the biopsy lesions ranged between 6 and 62 mm (average, 15.1 mm), with 3,451 (70.9%) lesions <15 mm and 1,416 (29.1%) lesions >15 mm (Table IB). Multiple lesions, either lateral or bilateral, were detected in 41.3% of the patients, with 25.1% harboring bilateral lesions (Table IB). A total of 43.0% of the lesions were localized in the upper external quadrant (Table IB).

### Clinicopathological features of patients and clinical outcomes

The histopathological examinations revealed that the majority of the lesions were benign [4,813/4,867 (98.89%)], with fibroadenoma [2,317/4,867 (47.61%)] and fibrocystic lesions [2,103/4,867 (43.21%)] appearing as the two most common pathological manifestations. A variety of other benign breast diseases, such as adenosis, mastitis, lipoma and intramammary lymph node were also observed (Table II). High-risk lesions, including papilloma, papillomatosis and atypical hyperplasia were apparent in 212 cases (4.36%; Table II). Fifty-four (1.11%) malignant lesions were detected among the specimens and a diagnosis was confirmed in all the cases. Complications occurred in a number of cases, including bleeding (843 cases, 22.9%), hematoma formation (1,012 cases, 27.5%) and scar formation (346 cases, 9.4%).

The mean follow-up period was 25.5 months (range, 1–60 months), during which time 162 patients (4.4%) were identified to have a recurrence (local recurrence, 77 patients; new lesion, 85 patients). Seventy-six of the 162 patients who were identified with local recurrence events or new lesions received re-excisions, and no second recurrence events (0/76) were observed.

## Discussion

To the best of our knowledge, a minimally invasive approach has almost replaced conventional open surgery in general surgery. It is even possible to treat well-selected gastrointestinal malignant diseases with minimally invasive surgery. To date, numerous studies have shown the efficacy and safety of minimally invasive surgery over open abdominal surgery and the advantages the minimally invasive approach presents, including improved cosmesis, less pain, early recovery and an enhanced quality of life (2–5,13,14). Traditionally, female patients with benign breast lesions underwent surgical excision; however, the availability of a minimally invasive approach has provided the option of a less aggressive and effective treatment, which may also be used in the general surgical field.

While open surgical biopsy possesses greater accuracy as a diagnostic tool, it is a more invasive procedure and involves the excision of a fragment of the breast parenchyma. As a result, it may lead to breast deformation and leave a scar. The development of minimally invasive techniques, including VABB, has provided an interesting alternative to open surgical biopsy in the diagnosis and treatment of focal lesions of the breast (16–18). VABB has been demonstrated to be well tolerated by patients, in addition to being efficient and correlated with relatively few complications. Its accuracy is reported to range between 98 and 100% (1) and is comparable with that of open surgical biopsy.

Breast carcinoma is the most common malignant tumor in female patients in China (14). The growing awareness of patients, the fear of cancer, the progress in imaging diagnostics and the relatively high availability of ultrasound examinations have required effective verification of nodular breast lesions. The differential diagnosis of small, nonpalpable lesions that are suspected of malignancy is particularly challenging. Until recently, the standard in such cases was open surgical biopsy. However, the potential complications, scarring and breast deformations, in addition to the duration of the procedure and the costs have resulted in efforts to investigate less invasive and cheaper methods. In the past 10 years, there has been substantial progress in the development of minimally invasive breast biopsy systems. As a type of minimally invasive method, VABB has been shown to be safe and effective for the definitive diagnosis and treatment of benign breast lesions (15). VABB is a minimally invasive procedure that is able to remove lesions under ultrasonography guidance, without repositioning or reinsertion. It demonstrated numerous advantages over surgical excision, including desirable cosmetic results, few complications, and patient convenience and satisfaction. Initially, VABB was used for biopsy; however, later, with advancements in the understanding of the technique, it has been used in attempts to excise lesions suspected to be benign tumors, such as fibroadenoma, fibrocystic lesions, adenosis and papilloma (15).

The Mammotome biopsy system is a minimally invasive surgical device that was introduced by Burbank *et al* in 1996 (19), successfully marketed by Ethicon Endo-Surgery, Inc. and approved by the US Food and Drugs Administration in 2004. The Mammotome biopsy system has been accepted as the standard for breast biopsy due to the fact that it provides an option to eradicate lesions using a minimally invasive approach. The Mammotome biopsy system procedure has become an effective and safe method for the complete excision of target lesions (20–23). Furthermore, it enables the biopsy to be performed in a visible and reliable manner, due to stereotactic, ultrasound and magnetic resonance guidance. The system probe is able to obtain an increased volume sample, resulting in a highly accurate and specific pathological diagnosis (1,20,24–27). Moreover, the cosmetic outcome achieved with the Mammotome biopsy system is able to satisfactorily meet the requisites of breast surgery and the acceptance of the patient. Therefore, the Mammotome biopsy system procedure has been highly recommended and has been applied increasingly in the treatment of benign breast lesions (21,22,27–30). Ultrasound guidance is currently widely applied due to the convenience it provides (21,22,27–30). In the present study, it was observed that the application of an 8-gauge ultrasound-guided VABB (Mammotome biopsy system) in the excision of presumed benign breast lesions resulted in high success rates, satisfactory cosmesis, good patient acceptance and few complications in a large sample.

Originally, the Mammotome biopsy system was guided stereotactically and was approved for breast biopsy with an 11-gauge probe (27). Following this, ultrasonography was used to offer real-time guidance and considerable progress was made due to a high-resolution linear transducer. Through the use of the Mammotome biopsy system, complete excisions have been achieved and the therapeutic management of benign breast diseases has developed incidentally (23). Increasing numbers of surgeons have attempted similar therapeutic procedures (21,27,28,30–32). The application of an 8-gauge probe led to the possibility of larger-volume sampling and excision (31). As a result of the potential for complete excision, biopsy and a treatment for benign breast lesions were facilitated. The clinical experience gained in the present study indicated that the 8-gauge probe was suitable for the firm breast tissue due to its sharp scalpel point, and that it was appropriate for the complete removal of benign lesions under ultrasound guidance (31). Taking into consideration the capacity of the probe to obtain the samples and to lead to successful therapeutic outcomes, the use of the 8-gauge probe was maintained throughout the study. The probe was convenient and efficient and resulted in few complications. Therefore, the outcome of the study has emphasized the therapeutic value of the 8-gauge probe in breast benign lesions. Notably, the advantages in the use of the larger 8-gauge probe in breast biopsy, compared with the 11-gauge probe, were disaffirmed (31). However, the 8-gauge probe was indicated to demonstrate greater potential in lesion eradication, rather than mere biopsy.

In a study involving 1,119 cases, in which the 8-gauge Mammotome biopsy system was successfully used to accomplish the complete excision of 2,163 lesions, the biopsy system was observed to be a feasible and effective method of managing benign breast diseases (14). In the present study, which involved 4,867 procedures in 3,681 patients, the management of benign and high-risk breast lesions with an 8-gauge Mammotome biopsy system was further indicated to be safe and effective. The rate of successful complete excision (99.7%) was higher in the present study than that in previous studies, which may have been due to the eradicative efforts that were enforced. However, we consider that there is a requirement for surgeons to avoid excessive excision, based on their experience.

In the current study, it was observed that patients commonly presented with multiple lesions, either lateral or bilateral (Table I). In these patients, ultrasound-guided VABB procedures using an 8-gauge Mammotome biopsy system have been indicated to be preferable over surgical procedures in which cosmetic damage is likely to occur, either due to scarring or excessive tissue excision. In the present study, 41.3% of patients presented with multiple lesions, of which 25.1% of the lesions were bilateral (Table I). The use of ultrasound-guided VABB with an 8-gauge Mammotome biopsy system in this subpopulation resulted in a satisfactory cosmetic outcome and patient acceptance. Therefore, the 8-gauge Mammotome biopsy system procedure, was suggested to be an appropriate first choice method for the excision of multiple benign or high-risk breast lesions.

A total of 54 malignancies were revealed in the present patient population. Interestingly, the majority of these cases were early breast cancers (Table II). Of note is the fact that, currently, the ultrasound-guided VABB procedure is not able to entirely replace surgery for breast cancer, although the complete excision of malignant lesions may be achieved. However, controversy remains with regard to high-risk lesions, such as papilloma, papillomatosis and atypical hyperplasia. For these lesions, subsequent surgery is not mandatory; however, intensive clinical and radiological supervision is necessary.

In the patients treated with ultrasound-guided VABB procedures in the present study, the most frequently occurring complications included hematoma, which occasionally required emergency surgical intervention. The most effective method of preventing bleeding from the surgical biopsy site was considered to be the application of a pressure dressing during the initial 24 h. This also prevented pain that may have been associated with the bleeding and the growing hematoma inside the breast.

There have been relatively few studies focusing on the recurrence of breast benign diseases following ultrasound-guided VABB. Grady *et al*(33) observed that the overall recurrence rate in patients with percutaneous excisions of fibroadenoma was 15%, following a median follow-up of 22 months. In the experience gained in the present study, it was noted that patients often made enquiries with regard to the rate of recurrence of their benign disease following complete excision. To the best of our knowledge, there have been relatively few studies concerning the clinical outcomes of patients with benign diseases following excision. In studies that have provided such data, the overall recurrence rate has ranged from 15 to 39% (32–35). The recurrence rate in the present study (4.4%) was markedly lower than that of previous studies. All the Mammotome probes used in the present study were 8-gauge, whereas some of the previous studies utilized 11-gauge probes. The lower recurrence rate in this study may have been due to the improved lesion clearance using the larger 8-gauge core.

The current study also focused on the pattern of recurrence in patients with benign breast lesions. There were 77 and 85 patients with local recurrences and new lesions, respectively. Our data revealed that patients with local recurrences tended to have a single lesion, of a larger size, when compared with patients with new lesions. A larger lesion size may increase the likelihood of residual lesions remaining, following ultrasound-guided VABB. Therefore, it was proposed that local recurrences occurring at the same site as that of the original lesion resulted from the regrowth of the residual lesion, due to incomplete excision. By contrast, patients with new lesions tended to have multiple lesions when compared with patients with *in situ* recurrence. Grady *et al*(33) suggested that the recurrence was the result of the growth of those small lesions, rather than incomplete re-excisions, due to the fact that the ultrasound examinations did not reveal any residual lesions following the initial excisions. However, it was considered in the present study that the microscopic residual lesions resulting from an incomplete excision may not always be detected by ultrasound. It was therefore considered that a local recurrence may still result from an incomplete excision.

In conclusion, the present study, which contained a large sample of ultrasound-guided VABB procedures, demonstrated that ultrasound-guided VABB using the Mammotome biopsy system (8-gauge) is a safe, effective, well-tolerated and minimally invasive procedure for the complete removal of benign breast lesions. Furthermore, the system was demonstrated to be able to rapidly remove the majority of benign breast lesions using a small incision and without the occurrence of scarring or complications. When microscopic examination reveals a benign lesion that corresponds to the clinical presentation, surgery may be avoided.

## Figures and Tables

**Table I:** Patient data and lesion characteristics.

A, Patient data (n=3681)	

Parameter	n (%)
Age (years)	
<35	1408 (38.3)
≥35	2273 (61.7)
BI-RADS category	
Category 2	682 (18.5)
Category 3	2150 (58.4)
Category 4	801 (21.8)
Category 5	48 (1.3)
Complications	
Bleeding	
None	2838 (77.1)
Small	589 (16.0)
Moderate/severe	254 (6.9)
Hematoma formation	
None	2669 (72.5)
Small	810 (22.0)
Moderate/severe	202 (5.5)
Scar formation	
None	3335 (90.6)
Small	302 (8.2)
Moderate/severe	44 (1.2)
Recurrence	
Local recurrence	77 (2.1)
New lesions	85 (2.3)

B, Lesion characteristics (n=4867)	
	
Parameter	n (%)

Lesion location in the inner breast	
Right breast	2287 (47.0)
Left breast	2580 (53.0)
Upper external quadrant	2093 (43.0)
Upper internal quadrant	876 (18.0)
Lower external quadrant	1411 (29.0)
Lower internal quadrant	487 (10.0)
Lesion size (mm)	
<15	3451 (70.9)
>15	1416 (29.1)
Lesion characteristic	
Non-palpable lesion	4195 (86.2)
Palpable lesion	672 (13.8)
Single lesion	2857 (58.7)
Multiple lesions	2010 (41.3)
Right lateral lesion	2005 (41.2)
Left lateral lesion	1640 (33.7)
Bilateral lesions	1222 (25.1)
Solid	3810 (78.3)
Cystic or mixed	1057 (21.7)
Complete removal of lesions	
Yes	4852 (99.7)
No	15 (0.03)

**Table II:** Histopathological characteristics (n=4867).

Histopathological diagnosis	n (%)
Benign	
Fibroadenoma	2317 (47.61)
Fibrocystic lesions	2103 (43.21)
Adenosis	54 (1.10)
Mastitis	63 (1.30)
Lipoma	44 (0.90)
Intramammary lymph node	4 (0.08)
Mammary ductal ectasia	8 (0.16)
Subcutaneous foreign body granuloma	8 (0.16)
Total	4601 (94.53)
High-risk	
Papilloma	56 (1.15)
Papillomatosis	54 (1.11)
Atypical lobular hyperplasia	83 (1.71)
Atypical ductal hyperplasia	19 (0.39)
Total	212 (4.36)
Malignant	
Ductal carcinoma *in situ*	21 (0.43)
Lobular carcinoma *in situ*	19 (0.39)
Infiltrating ductal carcinoma	10 (0.21)
Infiltrating mucinous carcinoma	4 (0.08)
Total	54 (1.11)
